# A sulfur and nitrogen cycle informed model to simulate nitrate treatment of reservoir souring

**DOI:** 10.1038/s41598-019-44033-5

**Published:** 2019-05-17

**Authors:** Moein Jahanbani Veshareh, Hamidreza M. Nick

**Affiliations:** Danish Hydrocarbon Research and Technology Centre, Technical University of Denmark, Lyngby, Denmark

**Keywords:** Environmental biotechnology, Carbon cycle

## Abstract

Nitrate treatment has been widely used in various seawater injection projects to treat biologic sulfate reduction or reservoir souring. To design a promising nitrate treatment plan, it is essential to have a comprehensive understanding of reactions that represent the microbial communities of the reservoir and mechanisms through which the souring process is inhibited. We employ a new approach of evaluating different reaction pathways to design reaction models that reflect governing microbial processes in a set of batch and flow experiments. Utilizing the designed models, we suggest dissimilatory nitrate reduction to ammonium is the main reaction pathway. Additionally, we illustrate nitrite inhibition is the major mechanism of nitrate treatment process; independent of nitrate reduction being autotrophic or heterotrophic. We introduce an inhibitory nitrate injection concentration that can inhibit souring regardless of nitrite inhibition effect and the distance between injection and production wells. Furthermore, we demonstrate that the ratio of the nitrite-nitrate reduction rate can be used to estimate nitrate treatment effectiveness. Our findings in regard to importance of nitrite inhibition mechanism and the inhibitory nitrate concentration are in accordance with the field observations.

## Introduction

While seawater injection in oil reservoirs is considered one of the most successful recovery methods, in some cases, high concentrations of sulfate leads to microbial induced reservoir souring by sulfate reducing bacteria (SRB), a process in which some amount of hydrogen sulfide (H_2_S) appears in the producing fluid from a reservoir that was initially sweet^1,2^. H_2_S can cause notable health and environmental risks^3^, and its corrosive nature, by decreasing the life expectancy of injection/production facilities, increases both capital and operational costs of oil and gas projects^4,5^. Therefore reservoir souring mitigation is highly desirable for oil companies.

One of the reservoir souring treatment strategies is nitrate treatment (NT)^6^. Addition of nitrate in injecting water can inhibit reservoir souring through four reported mechanisms. The first mechanism of NT is stimulation of nitrate-reducing bacteria (NRB). Since NRB and SRB (to reduce nitrate and sulfate) may use similar organic components as the electron donors, NRB can outcompete SRB for carbon sources^7^. The second mechanism of NT is to change the metabolism of SRB from sulfate reduction to nitrate reduction^8,9^. The third mechanism is stimulation of nitrate-reducing sulfide-oxidizing bacteria (NRSOB). NRSOB utilize nitrate or nitrite in order to oxidize sulfide to elemental sulfur or sulfate^10^. The fourth mechanism is inhibition of SRB by nitrate reduction products such as nitrite^11^.

Over the last decades, due to the wide utilization of NT, different numerical models have been utilized to simulate this process. Coombe *et al*.^12^ employed STARS reservoir model to simulate an experimental continuous up-flow packed-bed bioreactor data of Hubert *et al*.^13^ Coombe *et al*.^12^ model assumes that nitrate is directly reduced to N_2_ and sulfide is oxidized to sulfate. Hagshenas *et al*.^14^ used UTCHEM simulator in order to simulate experimental work of Reinsel *et al*.^15^ Haghshenas *et al*.^14^ assumed only a reaction through which nitrate is reduced to nitrite (no reaction was considered for nitrite reduction and no reaction was considered for sulfide oxidation). Cheng *et al*.^16^ used TOUGHREACT to simulate experimental work of Engelbrektson *et al*.^17^ They considered denitrification pathway for nitrate reduction by NRB or NRSOB and they assumed sulfide is only oxidized to sulfate. However, to the best of our knowledge, no research work has considered dissimilatory nitrate reduction to ammonium (DNRA) and sulfide oxidation to sulfur in simulation of the nitrate treatment process.

Vigneron *et al*.^18^ reported the water composition of various production wells in the Halfdan oil field. This data postulates that in the studied production wells there is an inverse relationship between nitrate and ammonium concentration, i.e. if the nitrate concentration is high then the ammonium concentration is low (and the opposite holds true if the ammonium is high). This can be due to reduction of nitrate to nitrite and then reduction of nitrite to ammonium. In another study on the Halfdan oil field, Gittel *et al*.^19^ claimed that the difference between ammonium concentration in the injection water (<0.01 mM) and the production water (2.3 mM) is due to dissimilatory nitrate reduction to ammonium (DNRA). However, observation of ammonium in production water samples cannot be regarded as sole evidence for DNRA, since ammonium in oil reservoirs can be derived from abiotic origins such as presence of clay minerals and/or alkali-feldspars^20,21^. Brunet and Garcia-Gill^22^ illustrated that sulfide inhibits the last two reduction steps of the denitrification process; therefore, the nitrate reduction pathway depends on the presence of sulfide. That is, if sulfide is present in the system, nitrate reduction occurs through the DNRA pathway. Additionally, Tiedje^23^ showed that in environments with high carbon source concentration available compared to nitrate (C/N), DNRA pathway is the dominant nitrate reduction pathway. As these conditions (presence of sulfide, high C/N) are likely to happen in nitrate treatment of reservoir souring, it is important to consider the DNRA pathway in simulations of reservoir souring.

In the past years, an excessive corrosion have been reported where nitrate was added in produced water reinjection facilities^24–26^. These reports are in agreement with laboratory experiments in which nitrate was added to mixed microbial cultures^27–29^. Since elemental sulfur (S0) is a strong oxidant of iron^30–32^, formation of S^0^, which is an intermediate product of sulfide oxidation by NRSOB, is speculated to be a cause of the excessive corrosion^25,27,29^. Therefore, in simulation of nitrate treatment process, it is important to consider sulfide oxidation to S^0^.

**Figure 1:**
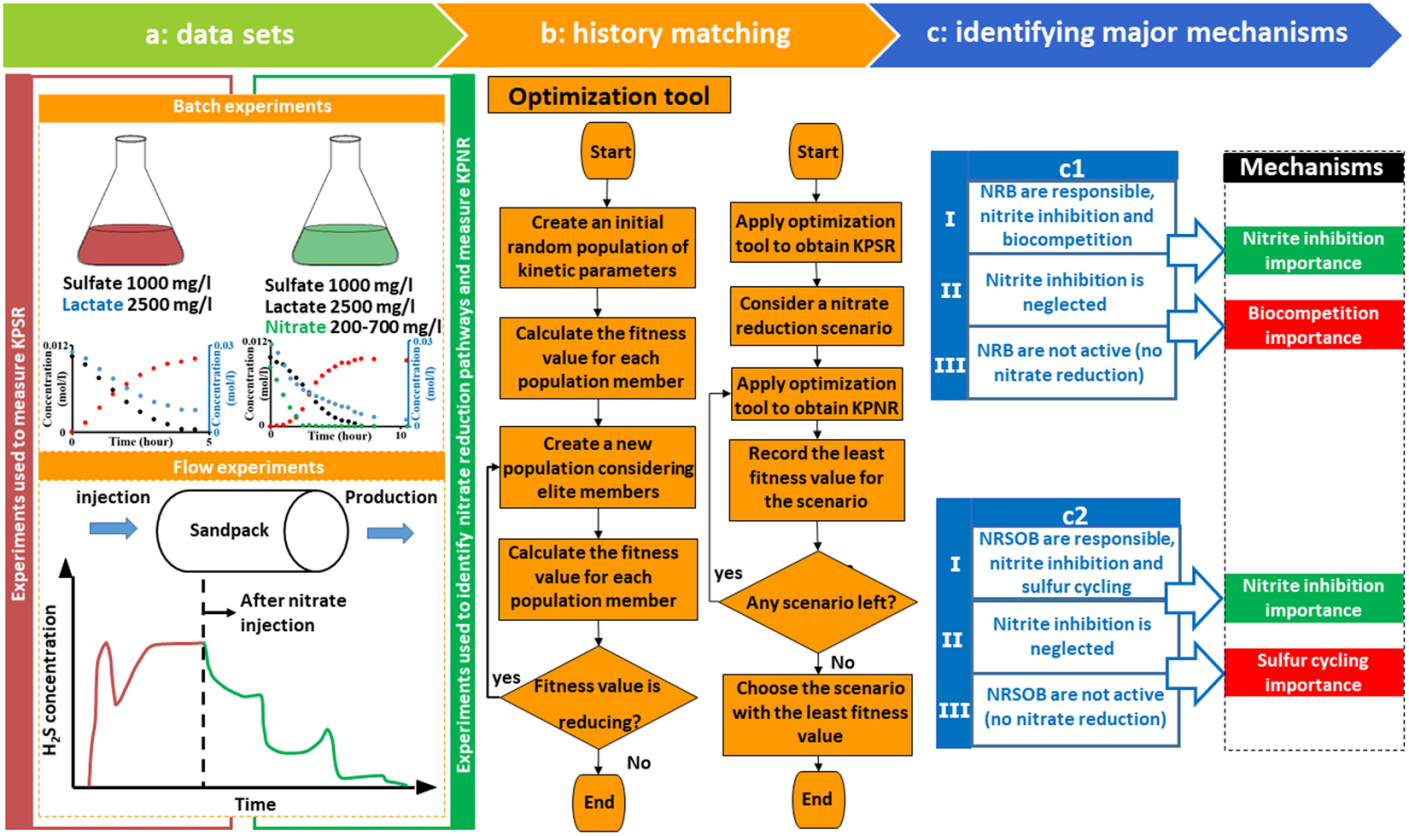
Schematic representation of data sets and methods used in this study. (**a**) A set of batch and flow experiments including only sulfate reduction (red) and, simultaneous sulfate and nitrate reduction (green). (**b**) The curve fitting algorithm utilized to obtain kinetic parameters of sulfate reducers (KPSR) as well as kinetic parameters of nitrate reducers (KPNR) (**c**) Different simulations designed to determine the relative importance of different mechanisms in nitrate treatment of reservoir souring.

In this work, we seek to develop a reaction model and a reactive transport model by utilizing the set of batch experiments from Xu *et al*.^33^ and the set of souring data from flow experiments by Hubert *et al*.^13^ Fig. 1 shows a brief overview of the workflow presented here. First, data from batch experiments are correlated, in which the types of responsible microorganisms are unknown. To do so, we employ a genetic algorithm to predict the most probable type of bacteria responsible for nitrate reduction as well as the most probable nitrate and sulfate reduction pathways. In the case of nitrate reduction, we evaluate whether nitrate is mainly reduced through the denitrification pathway or the DNRA pathway. In the case of sulfate reduction, we examine if sulfate is directly reduced to sulfide, or if it is first reduced to elemental sulfur and then sulfide. Then, the flow experiment data in which the category of responsible microorganisms is known (by microbiological tests) is correlated. To do this, we tune growth parameters for the category using reactive transport modeling. Afterward, model simulations are conducted to answer the following questions:

1. What is the dominant mechanism of NT processes?
2. What is the effect of flow path length on the required nitrate injection concentration?
3. What is the effect of nitrite reduction rate to nitrate reduction rate ratio on a NT process efficiency?
4. How much nitrate should be injected in the absence of nitrite inhibition effect to inhibit reservoir souring?

Answering these questions helps to establish some guidelines to assess NT processes. Subsequently, for different cases we can predict whether or not a NT process is effective, given that the type of microorganisms and their growth rate parameters are known. In the end, we compare our simulation results to two field reports. The first report shows NO_2_^−^ and H_2_S concentrations in different wells of a reservoir in the North Sea. The second report includes the history of NO_2_^−^ and H_2_S concentrations for a well in the Medicine Hat Glauconitic C field.

## Results

To simulate the batch experiments, different batch models each corresponding to a scenario (defined and described in the methods section, listed in Table S1) are assessed based on how accurate they can fit the experimental data, i.e. how small is their Sum of square errors (SSE).

The least SSE (Table S1) is calculated for the simulation results of a batch model corresponding to a scenario that only considers group A (a group representing heterotrophic nitrate and nitrite reduction through DNRA pathway). In order to compare batch models, the relative difference between SSE of each batch model (in percent) with the least SSE is calculated through RSSE_i_ = ((SSE_i_ − least SSE)/least SSE) × 100, where RSSE_i_ is the relative SSE for the i^th^ batch model.

**Figure 2:**
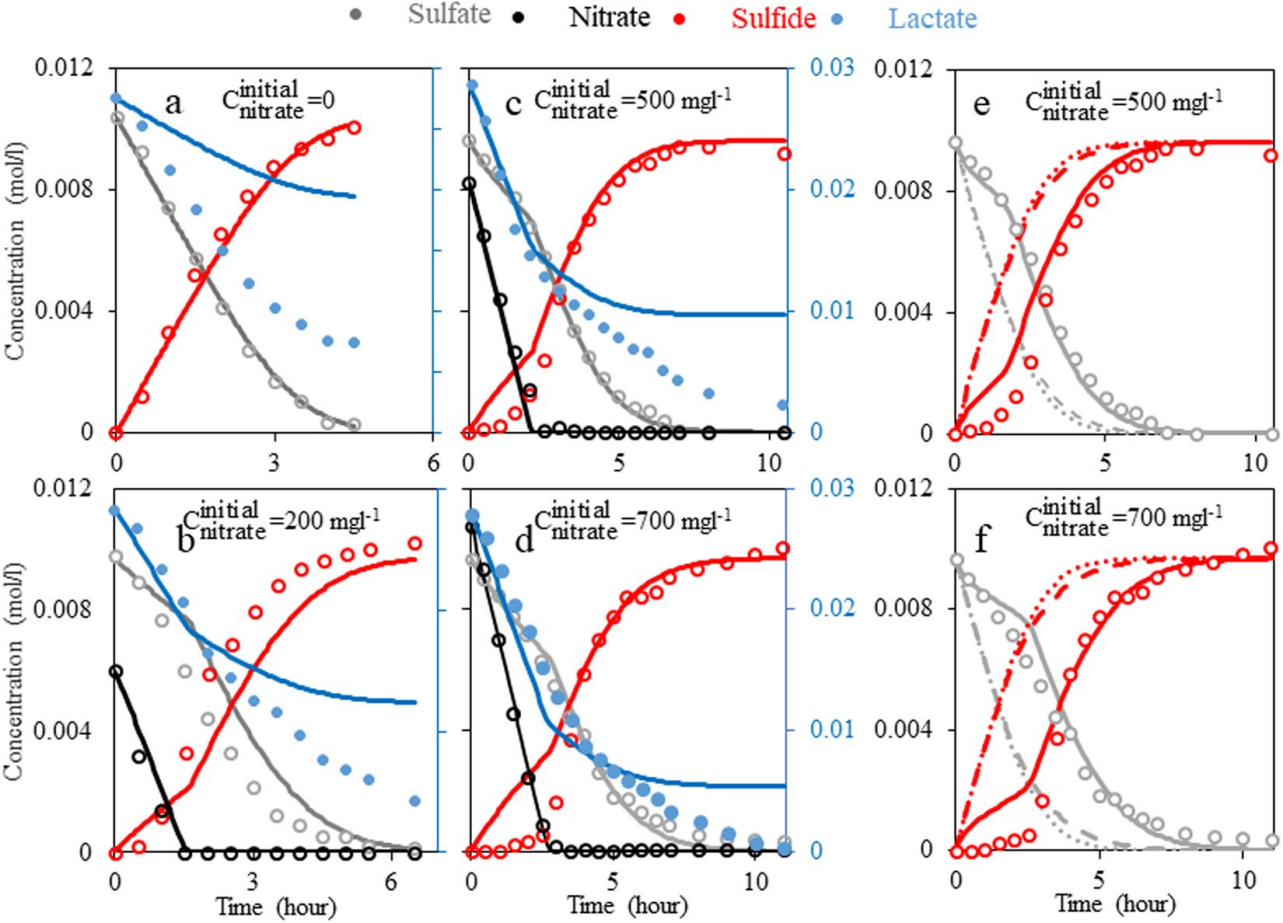
(**a–d**) Profiles of nitrate, sulfate, nitrite and lactate for different initial nitrate concentrations. Experimental data are shown by markers and simulation results (SIM1) are shown by solid lines. (**e**,**f**) Compare sulfide and sulfate concentrations measured with SIM1, SIM2 (SIM1 without considering inhibition effect, shown by dashed lines) and SIM3 (SIM1 without considering NRB activity, shown by dotted lines). Note that lactate concentration should be read from the right vertical axis, for other components concentration should be read from the left axis.

Except the scenario that only considers group A (the first scenario in Table S1), scenarios including group A with other groups (scenarios 2 to 6) have significantly lower SSE values compared to scenarios excluding group A (7 to 14). This observation suggests that heterotrophic nitrate/nitrite reduction (derived by NRB) through the DNRA pathway is the major responsible mechanism of nitrate reduction, and the presence of NRSOB and the denitrification pathway is not significant. Note that the genetic algorithm in this work is constrained such that it can minimize a group effect, but it cannot remove the effect. That is, in a case where a group called α is responsible, the optimization considering group α and another group leads to a SSE value close to, but not the same as, a SSE value where only α is considered. Therefore, a slightly different SSE value of the scenario considering group A only (scenario 1 in Table S1) than the SSE value of other scenarios including group A (alongside other groups, scenarios 2 to 6) do not result in a similar probability, SSE. Figure 2a–d show the batch experiments data and simulation results corresponding to the most probable case (referred to as SIM1; the batch model that considers the 1^st^ scenario of Table S1, Fig. 1c1). In the case of sulfate, sulfide, nitrate and nitrite, SIM1 prediction is reasonable (Table S2 lists parameters of SIM1).

It should be noted that, SIM1 cannot predict lactate trends with good precision. Figure 2b–d illustrate decline in lactate concentration at zero nitrate, nitrite and sulfate concentration, i.e. lactate consumption happens without presence of any electron acceptor. Thus, occurrence of fermentation (in addition to SRB, NRB and NRSOB activity) may be a reason why the current model cannot fit the lactate concentration data (as SIM1 does not consider any reaction for fermentation).

Notable differences among SSE of various scenarios (reported in Table S1) suggest that the classifications of microbial communities in the reservoir, and their metabolic pathways significantly influence the accuracy of simulations to predict reservoir souring, and effectiveness of nitrate injection. Additionally, it shows that by using a genetic algorithm, a simple batch experiment data set can be used to predict nitrate reduction pathways.

### What is the dominant NT mechanism by NRB

To illuminate the effect of nitrite inhibition, a new simulation is considered (referred to as SIM2, Fig. 1c1) and is fitted to the experimental results. SIM2 is SIM1 without the inhibition coefficient, i.e. all parameters are the same as SIM1 except that the inhibition coefficient, I/(C_Nitrite_ + I), is removed. Thereafter, to study the effect of biocompetition, NRB reactions are removed (a model that only contains SRB reactions with kinetic parameters the same as SIM1, referred to as SIM3, Fig. 1c1). In other words, SIM2 is SIM1 without nitrite inhibition effect and SIM3 is SIM1 without nitrite inhibition and biocompetition effects. Figure 2e,f demonstrate sulfate and sulfide concentrations for these three simulations (SIM1, 2, 3) compared to the experimental data. We also calculated SSE for sulfide and sulfate (reported in Table S3). From Fig. 2e,f and Table S3 it is clear that SIM2 is much less accurate and its deviation from experimental data increases by an increase in initial nitrate concentration. SIM3 predicts approximately the same concentration profile for sulfide and sulfate as that of SIM2. Table S3 data confirms this interpretation since RSSE (for sulfate and sulfide) for SIM2 and SIM3 are about the same (about 4% relative difference), and markedly different from SIM1. This implies that in experiments by Xu *et al*.^33^, nitrite inhibition is the major mechanism that suppresses SRB activity, whereas, biocompetition for a common carbon source does not have notable inhibitory effects. Therefore, we argue that if nitrite inhibition is not significant, and NRB are the only responsible community for nitrate treatment, nitrate treatment will not be effective.

### Simulation of the flow experiments

In the previous sections it was found that nitrite inhibition is the main mechanism through which heterotrophic nitrate and nitrite reduction through DNRA pathway can inhibit SRB activity in small scale batch experiments of Xue *et al*.^33^. Simulation of the batch experiments leaves two questions unanswered.

1. What is the nitrate treatment major mechanism if nitrate reduction occurs through an autotrophic process (rather than a heterotrophic)?
2. What is the interplay between reactive processes and flow?

To answer above mentioned questions we use the flow experiments of Hubert *et al*.^13^ where nitrate treatment using autotrophic NRSOB was evaluated through 1D domain flow experiments.

**Figure 3:**
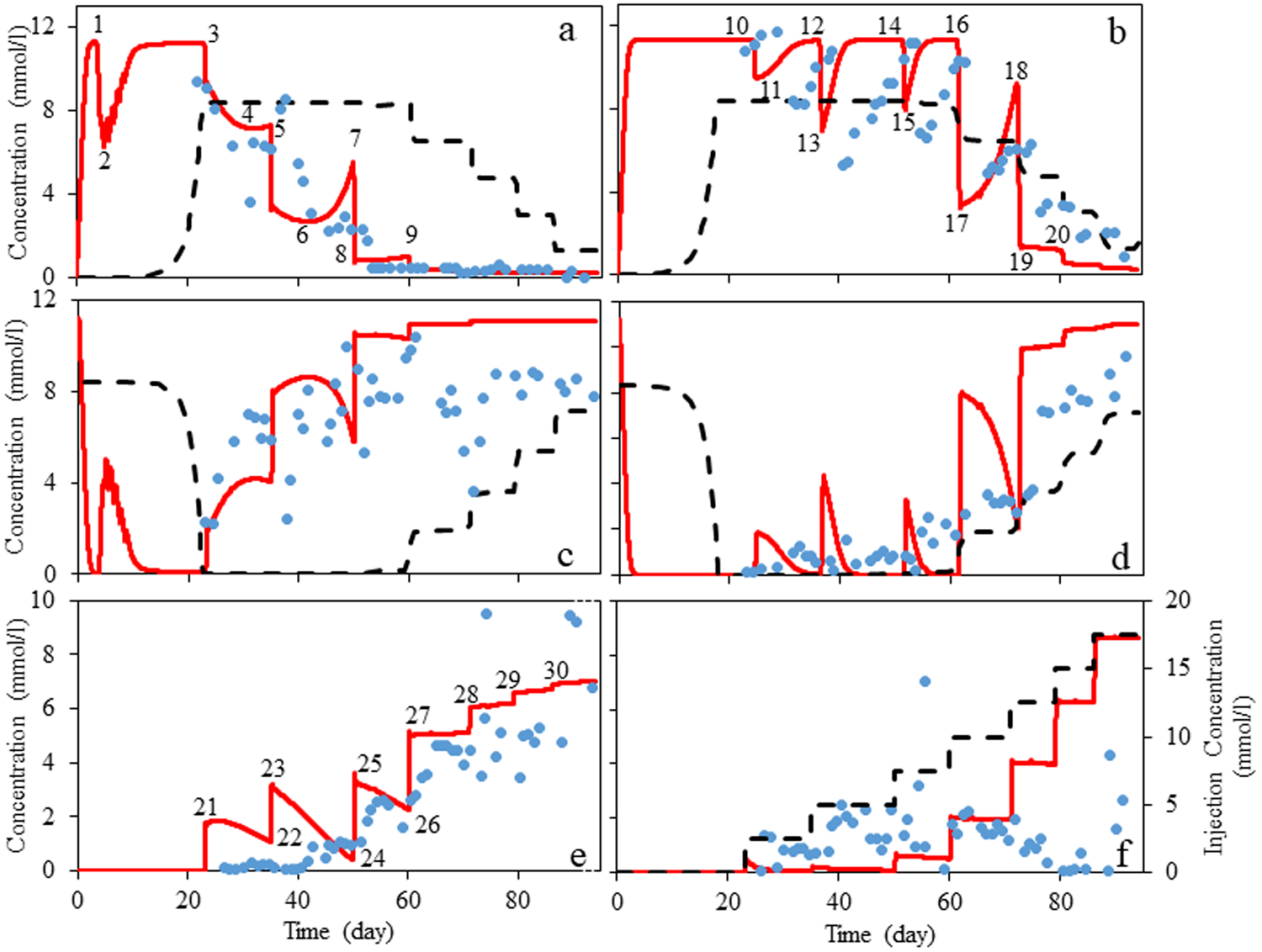
Comparison between data of the flow experiments (blue markers), simulation of the Coombe *et al*.^12^ (the dashed line) and authors’ model (the solid line). (**a**) Sulfide concentration, 1^st^ port; (**b**) sulfide concentration, 5^th^ port; (**c**) sulfate concentration, 1^st^ port; (**d**) sulfate concentration, 5^th^ port; (**e**), nitrate concentration, 1^st^ port; (**f**) nitrite concentration, 1^st^ port (the dotted line should be read from the right axis and shows nitrate injection concentration).

Figure 3 shows the simulation results of the reactive transport model (described in the method section) fitted to results of the flow experiments using the genetic algorithm (referred to as SIM4; Table S4 shows SIM4 model parameters, Fig. 1c2). Note that the only parameters that are fitted here are kinetic parameters of the reactions (a reaction representing SRB, and two reactions representing group B, Table S5).

Assuming conservation of mass, any consumption or production of sulfate is coincident with production or consumption of sulfide. That is, the simulation results for sulfide and sulfate in the first port (Fig. 3a,c), and the last port (Fig. 3b,d) are symmetric; therefore, explanations for changes (extremum values) observed in Fig. 3a,b, are also valid for changes observed in Fig. 3c,d. Main changes in sulfide and nitrate concentration trends are marked in Fig. 3 and are referred to as CH1 to CH30. Flowrate is initially zero until the complete reduction of sulfate happens (through reaction one), and then initial flowrate of 0.5 ml/h is applied. By imposing flowrate, nutrients pass biomass faster, so less reaction time leads to less production of sulfide, and more concentration of nutrients available for biomass. Therefore, a sharp drop in sulfide concentration occurs (CH1 to CH2, Fig. 3a), biomass starts to grow and sulfide production is gradually built up back to the previous level in a few days (CH2 to CH3, Fig. 3a). CH3 (Fig. 3a) is a result of the addition of 2.5 mM of nitrate at the inlet. Due to the introduction of nitrate, some sulfide is oxidized to sulfate (reactions 4 and 5 in Table S5) and leads to a rapid fall in sulfide concentration. After this rapid fall, two phenomena happen in parallel. First, growth of NRSOB leads to a higher degree of sulfide oxidation to sulfate. Second, since there is an excess amount of lactate in the system, SRB biomass grows to reduce the produced sulfate. These two phenomena proceed together. Initially, the sulfide oxidation rate is higher, so sulfide concentration is reduced (the trend between CH3 and CH4, Fig. 3a). However, by growth of NRSOB, nitrate and nitrite concentration, which reaches to the first port, is decreased. CH21 to CH22 (Fig. 3e) illustrates reduction in nitrite in the first port. Therefore, at CH4 (Fig. 3a) rate of sulfide reduction becomes equal to that of sulfide oxidation. Subsequently, sulfate reduction rate increases and causes an increase in sulfide concentration. The increase in sulfide concentration continues until the nitrate concentration at the injection point is increased. Again, the same behavior is observed; CH5 (Fig. 3a) is corresponding to increase of nitrate concentration from 2.5 to 5 mM. After a gradual decrease of sulfide oxidation rate (nitrite concentration that reaches the first port is decreased due to the growth of NRSOB, CH23 to CH24, Fig. 3e), and an increase of sulfate reduction rate, these two rates become equal at CH6 (Fig. 3a). Then, sulfate reduction continues to increase until the next change in nitrate concentration is applied (CH7, Fig. 3a). For the next increase in nitrate concentration, after the sharp decrease in sulfide concentration (CH7 to CH8, Fig. 3a), we cannot see the period in which sulfide oxidation rate is faster than sulfide reduction rate. A small increase in sulfide concentration is observed (CH8 to CH9, Fig. 3a) due to the reduction in the amount of nitrite that reaches the first port (CH25 to CH26, Fig. 3e). Increasing nitrate concentration at the injection point has a negligible effect on sulfide concentration in the first port for two reasons. First, nitrate concentration is high enough to oxidize all sulfide anions between the injection point and the first port. Second, nitrite concentration that reaches the first port is high enough to keep the sulfate reduction rate negligible. After the 60^th^ day, at nitrate injection concentrations more than 10 mM, nitrate and nitrite concentrations are no longer reduced (Fig. 3e,f). Since nitrate reduction is the result of sulfide oxidation, this means that for nitrate injection concentrations more than 10 mM no sulfate reduction (sulfide formation) occurs between the injection point and the first port.

Considering the 56 cm distance between the first and the last ports, every change in concentration at the first port is expected to occur at the last port approximately 1.55 days later (travel time of different components if convection is dominant and no retardation due to, for example, adsorption happens). The change in sulfide concentration due to increasing injection flow rate, as it was seen in the first port (CH1 and CH2, Fig. 3a), is not seen in the last port. This is because, the 56 cm distance is long enough for nearly all injected sulfate to be reduced before it reaches the last port, even for the greatest flow rate. The rest of the sulfide concentration changes observed in the first port (CH3 to CH9, Fig. 3a) are observed in the last port after 1.55 days (CH10 to CH18, Fig. 3b). That is, explanations given for CH3 to CH9, hold true for CH10 to CH18, respectively.

Coombe *et al*.^12^ also simulated the flow experiments using STARS simulator. In their work, for both ports, negligible souring is observed until about the 10^th^ day. It is not clear why souring is nearly zero in this timeframe. Regarding injection between 0–23 days, Coombe *et al*.^12^ do not show when the injection was applied. Their model gives a relatively good representation of the process for the last port; however, it cannot reasonably correlate the data for the first port. Considering the biological component of the sulfate reduction process and its inhibition by nitrate injection, concentration changes (in sulfate and sulfide) are expected due to growth of microorganisms (an exponential trend). Abrupt changes (rather than exponential) in sulfide and sulfate concentration profiles in the simulation result of Coombe *et al*.^12^ do not show this growth component. Additionally, models by Coombe *et al*.^12^ underestimate the effect of nitrate mitigation over most of the domain. This is probably because the effect of nitrite inhibition was neglected, and representative reactions for the governing biological processes were not considered.

### Impact of flow path length on the required nitrate injection concentration

Considering Fig. 3, nitrate injection concentrations in the range of 10 to 15 mM (corresponding to a timeframe between about the 60^th^ day and the 86^th^ day) can inhibit souring in the first port (Fig. 3a), but not in the last port (Fig. 3b). With these injection concentrations, a sufficient amount of nitrite is produced and present in the first port to inhibit SRB activity. However, due to the nitrite reduction between the first and last ports, nitrite concentration cannot be maintained between these ports. As a result, reservoir souring is inhibited in the first port but not in the last port. In higher nitrate injection concentrations (more than 15 mM, corresponding to a timeframe after the 86^th^ day) nitrite concentration is maintained at a reasonably high value throughout the column between the injection point and the last port.

**Figure 4:**
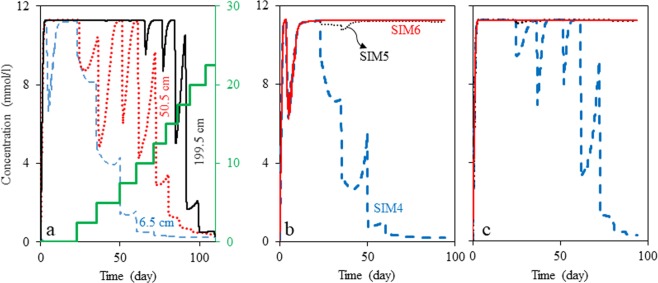
(**a**) Sulfide concentration at various locations of a 1D system, shows the dependency of required inhibitory nitrate concentration to the distance between injection and production wells; the solid green line shows nitrate injection concentration, and should be read from the right vertical axis. (**b**,**c**) Illustrates the effect of sulfur cycling and nitrite inhibition on the overall efficiency of the nitrate treatment strategy by NRSOB for the first and the last port, respectively. SIM4 is the simulation considering NRSOB reactions and nitrite inhibition effect, SIM5 is the simulation considering NRSOB reactions without inhibition effect, and SIM6 is the simulation without considering NRSOB activity.

In order to show the effect of flow path length more clearly, we changed the length of SIM4 model to 2 meters and conducted the simulation. The nitrate injection concentration in this simulation for the period of 94 to 110 days can be seen in Table S6. For this simulation, Fig. 4a shows sulfide concentration at 6.5 cm, 50.5 cm and 199.5 cm from the inlet. These sulfide concentration profiles for different distances demonstrate that nitrate injection concentration of 10 mM is enough to inhibit souring within a 6.5 cm distance from the injection point while 17.5 mM and 20 mM are needed for 50.5 and 199.5 cm distances. This suggests that when NT is applied to a subsurface reservoir, nitrate injection concentration should be set based on the distance between injection and production wells. That is, to design a successful NT process, nitrate injection concentration is recommended to be determined through simulation for different injection wells individually such that nitrate concentration is always maintained high enough throughout the medium between injection and production wells.

### Evaluation of NT mechanisms by NRSOB

In this section, SIM4 is used to explore the significances of different mechanisms of souring inhibition by NRSOB (nitrite inhibition and sulfur cycling). Initially, nitrite inhibition effect in the model is ignored (by removing the inhibition coefficient in eq. 1, this model is referred to as SIM5, Fig. 1c2), and then NRSOB activity is neglected (only considering SRB reaction, this model is referred to as SIM6, Fig. 1c2). The difference between the results of SIM4 and SIM5 represents the nitrite inhibition effect and the difference between the results of SIM5 and SIM6 illustrates the effect of sulfur cycling. Figure 4b,c indicate the results of these three models. SIM4 simulation results show that sulfide formation is decreased significantly as a result of nitrate injection. On the contrary, SIM5 results in a high sulfide concentration that is independent of nitrate injection. In fact, the results obtained by SIM5 and SIM6 have an approximately similar trend, indicating nitrate injection has a negligible effect on SRB activity. The significantly different trend of the results of SIM4 compared to those of SIM5 and SIM6 suggests that nitrite inhibition by NRSOB is a significant contributor to NT. The approximately similar trend of SIM5 and SIM6 results suggests that sulfur cycling by NRSOB is not a major contributor to NT.

Note that sulfur cycling can be effective only if the electron donor for SRB is limiting, and it cannot be effective if nitrate and nitrite are limiting. Considering the reaction representing the metabolism of SRB (Table S5, reaction 1), for each mole of lactate, 1.27 moles of sulfate reduces to sulfide. Therefore, in order to cause the system described in the flow experiments by Hubert *et al*.^13^ to run out of lactate, 31.87 moles of sulfate are needed. Regarding the sulfur cycling process, 31.87 moles of sulfate means that 31.87 moles of sulfide should be oxidized. The metabolism of NRSOB is described by reactions NRSOB1 and NRSOB2 (of Table S5), suggesting that each mole of nitrate can oxidize 0.29 moles of sulfide directly, and can oxidize 0.86 moles of sulfide indirectly (due to the production of nitrite). Thus, one mole of nitrate can oxidize 1.16 mole of sulfide; that is, in the absence of nitrite inhibition, ${\rm{1}}\mathrm{.10}\,(1\mathrm{.27}/1\mathrm{.16})$ moles of nitrate is needed to inhibit SRB activity in a hypothetical system. This hypothetical system assumes that:

1. One mole of lactate presents as the electron donor at the influx. Compared to real reservoir conditions, this continuous lactate influx is considered to account for organic matters that either initially occur in the formation brine (volatile fatty acids) or diffuse into the water phase from the oil phase, e.g. benzene, toluene, ethyl benzene and xylene.
2. NRSOB are nitrate and nitrite reducers only (there is no NRB).
3. Sulfide oxidization rate by NRSOB is equal to or greater than the sulfate reduction rate by SRB.

For example in the case of the flow experiments, considering presence of 25 mM of lactate in the system, regardless of the system length, 27.56 mM of nitrate should be present to inhibit SRB activity completely. This gives a general indication of how the Minimum Inhibitory Nitrate Concentration (MINC) can ensure SRB activity is inhibited. Note that MINC is dependent on the type and concentration of the electron donor, and it might be different for different reservoirs. However, the aim of introducing a 27.56 mM concentration of nitrate is to demonstrate that there can be a MINC, above which, souring can be completely inhibited. This nitrate concentration also shows that nitrite inhibition is significant, since it decreases the inhibiting nitrate injection concentration from 27.56 (MINC) to 15 mM. 15 mM is the nitrate concentration above which souring is inhibited in the presence of nitrite inhibition. We call this concentration the Required Inhibitory Nitrate Concentration (RINC).

Therefore, we suggest that in the flow experiments nitrite inhibition is the most important mechanism, in contrast with the interpretation of Hubert *et al*.^13^.

In regard with the flow experiment, firstly, although nitrite inhibition effect has been claimed to be not important, we suggest that nitrite inhibition is the most important mechanism. We also suggest that the RINC depends on the type and concentration of the carbon source, as well as the flow path length. That is, to have a successful nitrate treatment project, injecting the MINC is not necessary, and calculating the RINC for each individual injection well in an oil field can reduce nitrate consumption.

### Nitrite-nitrate reduction rate ratio

So far, the results highlight the importance of nitrite inhibition in NT, especially if the carbon sources in the system are in excess. Considering significant amounts of organic compounds either in the water phase or in the residual oil phase, along with the ability of microorganisms to utilize a vast spectrum of compounds as electron donors, nitrite inhibition effect on SRB appears to be a key factor for controlling the effectiveness of NT processes. The ratio of nitrite reduction rate to nitrate reduction rate (R) is one of the parameters that specifies concentration of nitrite in the system. Higher R values tend to lower nitrite accumulation, and in turn, lower NT efficiency. To show the importance of R on the souring inhibition capability of NRB and NRSOB, batch and flow simulations are conducted with a range of R values.

**Figure 5:**
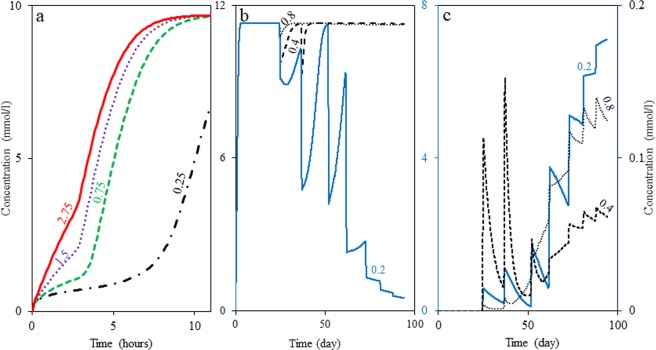
(**a**) Sulfide production under influence of NRB activity for various values of nitrite-nitrate reduction rate ratio (R) utilizing the model parameters of SIM1 with nitrate initial concentration of 700 mg/l. The solid, dotted, dashed and dashed-dotted lines are for R equals to 2.75, 1.5, 0.75 and 0.25, respectively. (**b**) Sulfide production affected by NRSOB activity for different values of R in the last port using the model parameters of SIM4. (**c**) Nitrate concentration corresponding to different cases of (**b**). In (**b**) and (**c**) the dotted, dashed, and solid lines are for R equal to 0.8, 0.4 and 0.2, respectively. Note that different R values are obtained by changing nitrite reduction rate.

To study souring inhibition capability of NRB at different R values, simulations are conducted with an initial nitrate concentration of 700 mg/l using parameters listed in Table S2. In order to set R, according to the maximum growth rate of NRB1, maximum growth rate of NRB2 is chosen to fulfill the desired range of R values. Figure 5a reveals that souring occurs at a slower rate if nitrite reduction happens at a slower rate than nitrate reduction.

To investigate the effects of R on the souring inhibition capability of NRSOB, simulations are run over 94 days using parameters listed in Tables S4 and S6. Similar to the approach used to evaluate NRB capability, maximum growth rate of NRSOB2 was chosen according to the maximum growth rate of NRSOB1 to span the desired range of R values. Figures 5b,c show sulfide and nitrite concentrations in the last port for these three simulations, respectively. They show that nitrate inhibitory effects observed for high R values are not significant. In other words, if NRSOB are the nitrate reducer community, R value for this community should be low in order to observe noticeable nitrate inhibition.

In summary, regardless of the type of nitrate reducers (NRB or NRSOB), R is an important factor that influences the success of nitrate mitigation strategies, i.e. If R is great (~1) for the nitrate reducer community of a reservoir, NT process for that reservoir seems to be not efficient.

### Comparison of simulation and field observations

**Figure 6:**
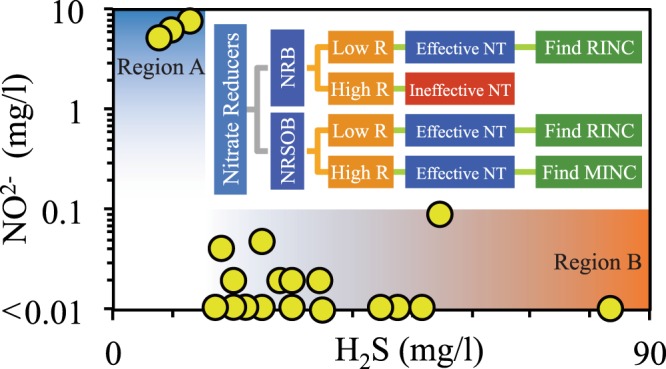
The relationship between NO_2_^−^ and H_2_S concentrations measured in several production wells of the Halfdan oil field reported by Vigneron *et al*.^18^. High H_2_S concentration is observed for low concentrations of NO_2_^−^ (region A). Significantly lower H_2_S concentration is observed for high concentrations of NO_2_^−^ (region B). Wells shown in region B are connected through direct fractures.

Figure 6 illustrates NO_2_^−^ and H_2_S concentrations in several production wells of the Halfdan oil field which have been subjected to NT^18^. This figure shows NO_2_^−^ concentration of wells for which nitrate treatment was not successful (great H_2_S concentrations), or was successful (small H_2_S concentrations). Therefore, it indicates a correlation between nitrate treatment success and NO_2_^−^ concentration. Utilizing our interpretations from the simulation results discussed in previous sections, and the data reported by Vigneron *et al*.^18^, we present two explanations for NO_2_^−^ and H_2_S concentration trends observed in those wells.

1. Insufficient nitrate has been injected through the injection well, i.e. nitrate injection concentration has been less than RINC or MINC. Therefore, nitrate and nitrite concentrations have become insignificant at some point between the injection and production wells. Subsequently, SRB activity or souring has remained untreated from that point to the production well (Region A in Fig. 6).
2. Sufficient nitrate has been injected, i.e. nitrate injection concentration has been equal or greater than RINC/MINC. Thus, nitrite concentration has been maintained between the injection and production wells. Due to the presence of nitrite inhibition along the entire distance between injection and production wells, SRB activity and in turn H_2_S concentration has been kept low (Region B in Fig. 6). We claim that nitrite inhibition has been the dominant mechanism because significant concentrations of volatile fatty acids (VFA) is observed in production water of wells in region B, meaning sulfur cycling and biocompetition could not have been effective. Note that, the distance between injector and producer wells in the Halfdan oil field is about the same. However, the three producer wells in region B of Fig. 6, are connected to the injectors through direct fractures. Presence of fracture reduces the travel time (time that nitrate and nitrite are exposed to nitrate reducers), and subsequently, the nitrate concentration required to prevent reservoir souring.

A similar trend (a reverse relationship between NO_2_^−^ and H_2_S concentrations) can be seen in Fig. 2 in the work of Agrawal *et al*.^7^ where nitrite concentration breakthrough is coincident with the end of H_2_S production. In the work of Agrawal *et al*.^7^, souring control and nitrite breakthrough is not obtained through increase in nitrate injection concentration, rather by a decrease in nitrite reduction due to the removal of toluene. This means that when nitrate injection is started, the concentration of NRB favorite electron donors is high. Thus, nitrite reduction happens after nitrate reduction (Fig. 3 in the work of Agrawal *et al*.^7^ shows that nitrite accumulation occurs if nitrate exists). However, as time proceeds there are not enough electron donors to reduce nitrite due to the dilution of electron donors. So nitrite is maintained and sulfide production is inhibited.

In 2004, for a field in the North Sea, Sunde *et al*.^34^ reported that changing the mitigation strategy from biocide injection to nitrate injection caused 1000 times decrease in SRB population, 50% decrease in observed corrosion, and lesser H_2_S production. The effectiveness of this NT process is cited in other works^35–38^. However, Mitchell *et al*.^39^ reinvestigated the same field in 2017 and discovered that the H_2_S level in the field for most of the wells increased after 2004. Therefore, Mitchell *et al*.^39^ suggested that the decrease in H_2_S production before 2004 was not due to nitrate treatment, concluding that NT did not have a desirable effect. Considering our simulation together with previous field reviews (Vigneron *et al*.^18^ and Agrawal *et al*.^7^), we argue that ineffectiveness of NT in the Gullfaks oil field reported by Mitchell *et al*.^39^ might not be due to the ineffectiveness of NT, but due to injecting an insufficient concentration of nitrate throughout the field.

## Methods

Figure 1 schematically shows data sets and methods that were employed in this work. Two experimental data sets are used in this work, a set of batch experiments and a set of flow experiments. Each of these two data sets consists of two parts. The first part is data corresponding to experiments that only include sulfate reducers activity (shown by red color in Fig. 1a). The second part is data corresponding to experiments that consider sulfate reducers activity along with nitrate reducers (shown by green color in Fig. 1a).

To simulate these data sets batch models and a reactive transport model are designed. In this section, first, we explain how batch models are developed. Each batch model is a reaction network that reflects a part of nitrogen cycle. Therefore, we illustrate how we represent the nitrogen cycle through different reaction networks. Then we describe a way to screen these reaction networks to find a reaction network that represents the batch experiments the best. Second, we characterize the reactive transport model developed to simulate the flow experiments.

### Batch experiments of Xu *et al*.^33^

We use the anaerobic part of Xu *et al*.^33^’s experiments (referred to as the batch experiments), in which simultaneous reduction of both nitrate and sulfate are reported, along with nitrite concentration throughout the experiments. We use the anaerobic part since oxygen is considered to be negligible in petroleum reservoirs. Five batch experiments were conducted. In each of these experiments the same initial sulfate and lactate concentration of 1000 mg/l and 2500 mg/l, respectively, and nitrate concentrations of 0, 200, 500, 700, or 1000 mg/l were applied (data reported in Fig. 1 in the work of Xu *et al*.^33^). A significant amount of biomass (8000 mg/l) was inoculated so that the biomass growth over the short period of the experiments (from 4.5 to 12 hours) could be ignored.

In order to model the batch experiments, eleven reactions are considered, each representing the metabolism of a microbial community. Each reaction is obtained by combining an electron donor half reaction, an electron acceptor half reaction and a cell synthesis half reaction following the bioenergetics concept^40^. In the supplementary material information required to obtain the reaction for each community is available (Tables S5 and S7).

**Figure 7:**
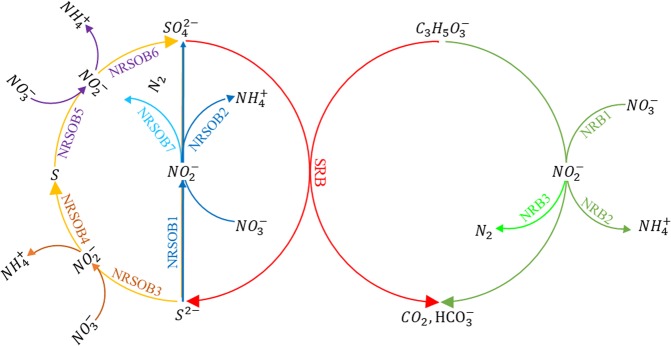
Schematic representation of the different reaction pathways for reduction of nitrate, nitrite and sulfate; different groups of (Table S5) are shown by different colors.

Only one community (reaction) is considered for sulfate reduction (SRB); however, since nitrate and nitrite reduction can happen through different pathways, ten communities (reactions) are considered for nitrate and nitrite reduction. The word community is used since maybe more than one strain derives the corresponding metabolism. Each community represents a part of the nitrogen and sulfur cycle as shown in Fig. 7. In fact, Fig. 7 consists of all half reactions listed in Table S7 (excluding the cell synthesis half reaction, half reaction number 8) that have been arranged regarding communities listed in Table S5.

Monod formulation is considered for the kinetics of each reaction (growth of each community):

1 $$r={\mu }_{max}{C}_{biomass}\frac{{C}_{d}}{{C}_{d}+{K}_{d}}\times \frac{{C}_{a}}{{C}_{a}+{K}_{a}}\times \frac{I}{{C}_{inhibitor}+I}-b{C}_{biomass}$$

where *r* (mol/l/s) is the reaction rate, *μ*_*max*_ (1/s) is the maximum growth rate, *C*_*d*_ (mol/l) is the electron donor concentration, *K*_*d*_ (mol/l) is the electron donor half saturation constant, *C*_*a*_ (mol/l) is the electron acceptor concentration, *K*_*a*_ (mol/l) is the electron acceptor half saturation constant, ${C}_{inhibitor}$ (mol/l) is the inhibitor concentration, *I* (mol/l) is the inhibition coefficient of the inhibitor and b is the endogenous decay coefficient (1/s). The inhibition parameter is applied only to take into account inhibition effects of nitrite on SRB activity; therefore, it is only present in the rate expression for SRB.

In order to evaluate which community/communities (pathway/pathways) is/are responsible for the nitrate and nitrite reduction, different possible combination of communities (referred to as scenarios, listed in Table S1) are examined. Based on the experimental results, the following two constraints are considered in the studied scenarios:

1. Nitrite is produced and then reduced; that is, in each scenario there has to be a nitrate reducer and a nitrite reducer.
2. The pair of reactions that utilize sulfur as the electron donor, cannot be present without presence of the reaction pair that oxidizes sulfide to sulfur.

To consider the first point, we group communities. Each group is composed of two communities that share a similar electron donor; a group reduces nitrate to nitrite and another group reduces nitrite to either ammonium or N_2_. Table S5 shows all of the examined groups and their corresponding communities. Groups A to D are derived based on the DNRA pathway (Fig. 7, community pairs that point to ammonium), and groups E and F are derived based on denitrification (Fig. 7, community pairs that point to N_2_). Groups A, E are representative of heterotrophic nitrate and nitrite reduction (NRB, shown by green color in Fig. 7). Group B, C, D, and F are considered for autotrophic nitrate and nitrite reduction (NRSOB). Groups B and F take into account nitrate and nitrite reduction, and sulfide oxidation to sulfate (blue color in Fig. 7). Group C includes nitrate and nitrite reduction, sulfide oxidation to sulfur (brown color in Fig. 7). Group D demonstrates nitrate and nitrite reduction, sulfur oxidation to sulfate (purple color in Fig. 7). Having the definition of each group, the pair of reactions that utilize sulfur as the electron donor is group D and the pair of reaction which oxidize sulfide to sulfur is group C. Therefore, the second constraint means that in none of the possible scenarios group D cannot be present without group C.

DNRA can occur through 11 different ways, since groups A to D can be combined in 15 forms, four of which include group D without C. In the case of denitrification, we do not consider sulfide reduction to sulfur and sulfur oxidation to sulfate, so only three scenarios are evaluated (groups E and F can be combined only in three ways). For more detail on how different scenarios are obtained, look at Table S8. Therefore, 11 + 3 scenarios (listed in table S1) can be responsible for the nitrate and nitrite reduction in the batch experiments. Each scenario in addition to SRB community is a reaction network, and makes a batch model. Each batch model has parameters corresponding to its reaction network. For example, if a reaction network contains α communities, then the batch model corresponding to this reaction network contains α set of kinetic parameters (defined in equation 1).

The batch experiments data include sulfate reduction (Fig. 1a (this work), red, Fig. 1a of Xu *et al*.^33^) and simultaneous sulfate and nitrate reduction (Fig. 1a, green, Fig. 1b–d of Xu *et al*.^33^). Therefore, the first part is used to measure kinetic parameters of sulfate reducers (KPSR). Having KPSR measured, the second part is used to find kinetic parameters of nitrate reducers (KPNR). Note that each scenario is characterized by a set of KPNR. To obtain parameters of each batch model (KPSR+KPNR), curve fitting is employed. That is, sum of square errors (SSE) between experimental data and each batch model output (residuals) is minimized. Figure 1b illustrates the minimization process. Minimization is done using the genetic algorithm function of MATLAB to find the global minimum. Briefly, a random initial population is created for KPSR or KPNR (within the range reported in the literature). Next, for each member of the population the difference between the model prediction and experimental data sets is calculated (the fitness value). Population members with lower fitness values are chosen as elite members. Elite members are utilized to make the next population. This iterative procedure is continued until a sufficient sulfate reduction model is obtained (where by changing KPSR or KPNR the model cannot fit the experimental data significantly better). Further refinement is obtained using MATLAB’s fmincon optimization function. Minimizations are constrained. Table S9 lists the constraints of the parameters. Additionally, since in the batch experiments 8000 mg/l of biomass was used, a linear constraint is used to keep the sum of biomass concentrations (of different communities) less than 8000 mg/l.

Curve fitting for each batch model results in a final SSE. Note that KPSR are the same for each batch model, therefore, curve fitting for finding KPSR is done only once. Using the fixed KPSR, multiple curve fitting is done to calculate the KPNR corresponding to each scenario. That is, SSE here, refers to SSE obtained through the minimization process to calculate KPNR. In other words, SSE of each batch model can represent how probable its corresponding scenario is responsible for the nitrate and nitrite reduction in the batch experiments. Therefore, the most significant batch model is the one that results in the lowest SSE, or the highest similarity with the batch experiments data. This batch model contains a reaction network corresponding to the scenario that most probably is responsible for the nitrate and nitrite reduction in the batch experiments.

The abovementioned batch models takes SRB inhibition into account only if nitrate and/or nitrite are/is present. In other words, when the medium runs out of nitrate and/or nitrite, SRB activity (reflected in the increase of sulfide and decrease of sulfate concentrations) in the model increases to the normal rate (the rate if nitrate treatment was absent). This is why none of the batch models can accurately predict data of Xu *et al*.^33^ in Fig. 1e or the data with initial nitrate concentration of 1000 mg/l (from Xu *et al*.^33^ study, after 6 hours, the medium runs out of nitrate and nitrite, yet sulfate and sulfide concentrations remain relatively unchanged). Therefore, by the batch experiments data, we refer to data reported in Fig. 1a–d of Xu *et al*.^33^.

### Packed-bed bioreactor experiment of Hubert *et al*.^13^

There are several experimental-based studies presented in the literature in which the souring process and mitigation through packed-bed bioreactors were investigated. We use the data reported by Hubert *et al*.^13^ in which sulfur cycling is considered to be caused by NRSOB; this work is called “the flow experiments” in the rest of the paper (Fig. 1a). The setup is consisted of glass column bioreactors with dimensions of 4.5 cm × 64 cm in which there are five equally spaced (14 cm spacing) sampling ports. The first port position is 6 cm from the bottom and the last port is 2 cm below the top of the flow cell. Bioreactors were filled with sand grains of size 225 µm to provide a medium for biofilm formation. Initially a medium containing sulfate and lactate was injected into the bioreactor followed by inoculation of produced water from a reservoir. Afterward, the system was operated batchwise until there was complete sulfate reduction in the system. Then, a medium containing sulfate and lactate was injected with an injection rate of 0.5 ml/h, and this rate was increased daily by 0.5 ml/h to reach to a final rate of 9 ml/hr. After 3 weeks, the injection fluid composition was changed by an addition of 2.5 mM of nitrate. The time table of sulfate, lactate and nitrate concentration applied in the influent is given in Coombe *et al*.^12^. However, in Coombe *et al*.^12^, different sulfate injection concentrations are considered (Table S6). Sulfate concentrations in Table S6 are based on mass conservation; that is, summation of sulfate and sulfide concentration throughout the glass column is conserved. Therefore, after each change in nitrate concentration, summation of sulfide and sulfate concentration was calculated for several consecutive data points and the average value was considered as the sulfate injection concentration.

In order to model the bioreactor column, it is considered as a 1D system discretized using a finite volume method into 64 numerical grids, and the average results of the 6^th^ and 7^th^ grids, and the 62^th^ and 63^rd^ grids were considered to compare with the first and last nodes of glass column bioreactors used in the flow experiments. Hubert *et al*.^13^ did not mention what sulfate concentration they regarded as complete reduction. Therefore, the sulfate concentration of 0.1 mM is considered since it corresponds to 99% sulfate reduction. To represent the procedure of the flow experiment, the initial fluid velocity of zero is considered in the media before the sulfate concentration reduces to 0.1 mM. Afterward, the fluid flow rate is increased each day by 0.5 ml/hr, and influent composition is changed according to Table S6.

Hubert *et al*.^13^ claimed that NRSOB are responsible for inhibitory effects of nitrate injection. However, they did not specify if the nitrate reduction pathway is DNRA or denitrification. After complete reduction of sulfate in the flow experiments, environment of the sand pack column is characterized by having a high sulfide concentration (~11 mM). Brunet and Garcia-Gil (1996) showed that presence of sulfide in the form of H_2_S suggests the nitrate reduction pathway to be DNRA. Therefore, reactions of group B in Table S5 are applied in the reaction engine of a reactive transport model to simulate the flow experiment.

### Reactive transport modeling

The reactive transport equation can be written in the following general form^41,42^:

2 $$\partial (\varphi {C}_{i})/\partial t={\nabla }\cdot (\varphi {D}_{i}{\nabla }{C}_{i})-{\nabla }\cdot (q{C}_{i})-{\sum }_{j=1}^{Nj}{v}_{ij}{R}_{j}$$

where *R*_*j*_ is aqueous phase reactions, *N*_*j*_ is total number of reactions that include component *i*, ${v}_{ij}$ is stoichiometric coefficient of component $i$ in the ${j}^{th}$ reaction, $\varphi$ is porosity, *D* is diffusion and dispersion coefficient, *C*_*i*_ is concentration of component *i* and *q* is Darcy velocity.

Note that zero diffusion and advection terms are considered for biomass of microorganisms. An operator-splitting method was used to develop our simulator. A detailed algorithm of this method can be found in the work of Parkhurst and Wissmeier^43^.

## Supplementary information

Supplementary Materials
