# Supplementary material for: A sulfur and nitrogen cycle informed model to simulate nitrate treatment of reservoir souring
Source: Sci Rep. 2019 May 17;9:7546. doi: 10.1038/s41598-019-44033-5 (PMC6525193; doi:10.1038/s41598-019-44033-5)
Supplement: Supplementary file 1 — Supplementary Materials [file 41598_2019_44033_MOESM1_ESM.pdf]

A sulfur and nitrogen cycle informed model to simulate nitrate treatment of reservoir souring

Moein Jahanbani Veshareh,[1,*] Hamidreza M. Nick[1]

[1]Danish Hydrocarbon Research and Technology Centre, Technical University of Denmark, Lyngby, Denmark

* Corresponding Author. Email: moein@dtu.dk

Supporting Information

7 Pages

7 Tables

Table S1. List of evaluated possible scenarios and their corresponding sum of square errors when they are fitted to the batch experiments

	Scenario/batch model	Involving groups	SSE*	RSSE*
DNRA	1	A (SIM1)	0.216	0
	2	AB	0.218	0.926
	3	AC	0.217	0.463
	4	ABC	0. 217	0.463
	5	ACD	0.258	19.44
	6	ABCD	0.266	23.15
	7	BC	0.479	121.76
	8	BCD	0.478	121.29
	9	B (SIM4)	0.476	120.37
	10	C	1.046	384.26
	11	CD	0.479	121.76
Denitrification	12	E	0.276	27.78
	13	F	0.458	112.04
	14	EF	0.279	29.17

*SSE: sum of square errors

**RSSE: relative difference of each SSE with the least SSE (in percent)

Table S2. SIM1 (the batch model) parameters corresponding to the best fit (the batch experiment of Xu et al.[1])

Kinetic parameters	SRB	NRB1	NRB2
μ (1/s)	2.952×10^{-3}	1.915×10^{-3}	5.288×10^{-3}
K_a (mol/l)	3.878×10^{-3}	4.546×10^{-5}	1.176×10^{-3}
K_s (mol/l)	5.105×10^{-3}	7.243×10^{-3}	7.544×10^{-3}
$C_{biomass\ initial}^{*}$ (mol/l)	5.374×10^{-3}	2.951×10^{-3}	1.805×10^{-2}
I (mol/l)	1.450×10^{-4}	-	-

Table S3. Effect of removing inhibition effect and NRB activity from the most probable model (SIM1)

	SSE for sulfate and sulfide	RSSE for sulfate and sulfide	The difference with SIM1
SIM1	0.082	0	-
SIM2	0.148	80.45	The inhibition coefficient in equation 1 is removed
SIM3	0.160	95.12	Nitrite inhibition and biocompetition are both removed (by considering μ=0 for NRB1 and NRB2)

Table S4. SIM4 (the reactive transport model) parameters (based on the flow experiment of Hubert et al.[2])

Kinetic parameters	SRB	NRSOB1	NRSOB2
μ (1/s)	1.2×10^{-3}	2.578×10^{-3}	1.576×10^{-3}
K_a (mol/l)	5×10^{-3}	2.592×10^{-3}	4.187×10^{-3}
K_s (mol/l)	1×10^{-3}	6.027×10^{-3}	5.911×10^{-3}
$C_{biomass\ initial}^{*}$ (mol/l)	7.5×10^{-4}	7.5×10^{-4}	7.5×10^{-4}
I (mol/l)	2.042×10^{-4}	-	-
b (1/s)	2.4×10^{-5}	5.156×10^{-5}	3.152×10^{-5}

Communities' reactions

Derivation of overall stoichiometric reaction for a microbially mediated reaction needs three oxidation half reactions, one for the electron donor (R_d), and two others for the electron acceptor (R_a) and bacterial cell synthesis (R_c). The overall reaction then is obtained through the following equation:

$$R=R_d-f_eR_a-f_sR_s \tag{1}$$

f_e is the fraction of electron donor which is coupled with the electron acceptor to produce energy (catabolism). f_s is the fraction of electron donor which is coupled with cell synthesis to produce biomass (anabolism).

For example to obtain the reaction for SRB we combine reactions 1, 6 and 8 (from Table S2) based on equation 1 as follow:

$$\left\{\frac{1}{12}CH_3CH(OH)COO^{-}+\frac{1}{3}H_2O\rightarrow\frac{1}{6}CO_2+\frac{1}{12}\ HCO_3^{-}+H^{+}+e^{-}\right\}-$$

$$0.85\times\left\{\frac{1}{16}H_2S+\frac{1}{16}HS^{-}+\frac{1}{2}H_2O\rightarrow\frac{1}{8}SO_4^{2-}+\frac{19}{16}H^{+}+e^{-}\right\}-$$

$$0.15\times\left\{\frac{1}{20}\ C_5H_7O_2N+\frac{9}{20}H_2O\rightarrow\frac{1}{5}CO_2+\frac{1}{20}HCO_3^{-}+\frac{1}{20}NH_4^{+}+H^{+}+e^{-}\right\}$$

The result will be the following reaction:

$$\frac{1}{8}SO_4^{2-}+\frac{255}{1600}H^{+}+\frac{15}{2000}NH_4^{+}+\frac{1}{12}CH_3CH(OH)COO^{-}\rightarrow\frac{15}{2000}$$

$$C_5H_7O_2N+\frac{41}{300}CO_2+\frac{91}{1200}\ HCO_3^{-}+\frac{85}{1600}H_2S+\frac{85}{1600}HS^{-}+\frac{955}{6000}H_2O$$

Table S5. List of half reactions for each community including f_e and f_s

Pathway	Reaction number	Group	Community	Electron donor	Electron acceptor	Cell synthesis half reaction (R_s)	Electron donor half reaction (R_d)	Electron acceptor half reaction (R_a)	f_e	f_s
	1	-	SRB	Lactate	Sulfate	8	6	1	0.85	0.15
DNRA	2	A	NRB1	Lactate	Nitrate		6	2	0.46	0.54
	3		NRB2	Lactate	Nitrite		6	3	0.49	0.51
	4	B	NRSOB1	Sulfide	Nitrate		1	2	0.85	0.15
	5		NRSOB2	Sulfide	Nitrite		1	3	0.87	0.13
	6	C	NRSOB3	Sulfide	Nitrate		7	2	0.79	0.21
	7		NRSOB4	Sulfide	Nitrite		7	3	0.81	0.19
	8	D	NRSOB5	Sulfur	Nitrate		5	2	0.86	0.14
	9		NRSOB6	Sulfur	Nitrite		5	3	0.88	0.12
Denitrification	10	E	NRB1	Lactate	Nitrate		6	2	0.46	0.54
	11		NRB3	Lactate	Nitrite		6	4	0.33	0.77
	12	F	NRSOB1	Sulfide	Nitrate		1	2	0.85	0.15
	13		NRSOB7	Sulfide	Nitrite		1	4	0.76	0.24

Table S6. Time table of sulfate, lactate and nitrate concentration in the influent, used in reactive transport models

Time range (days)	Lactate (mM)	Sulfate (mM)	Nitrate (mM)
0.0-23	25	11.3	0
23-35			2.5
35-50			5.0
50-60			7.5
60-71			10.0
71-79			12.5
79-86			15.0
86-94			17.5
94-104			20
104-110			22.5

Table S7. Half reactions used together with Table S5 to develop reactions representing metabolism of different microbial communities

1	$\frac{1}{16}H_2S+\frac{1}{16}HS^-+\frac{1}{2}H_2O\rightarrow\frac{1}{8}SO_4^{2-}+\frac{19}{16}H^++e^-$
2	$\frac{1}{2}NO_2^-+\frac{1}{2}H_2O\rightarrow\frac{1}{2}NO_3^-+H^++e^-$
3	$\frac{1}{6}NH_4^++\frac{2}{6}H_2O\rightarrow\frac{1}{2}NO_2^-+\frac{8}{6}H^++e^-$
4	$\frac{1}{6}N_2+\frac{2}{3}H_2O\rightarrow\frac{1}{3}NO_2^-+\frac{4}{3}H^++e^-$
5	$\frac{1}{6}S+\frac{2}{3}H_2O\rightarrow\frac{1}{6}SO_4^{2-}+\frac{4}{3}H^++e^-$
6	$\frac{1}{12}CH_3CH(OH)COO^-+\frac{1}{3}H_2O\rightarrow\frac{1}{6}CO_2+\frac{1}{12}HCO_3^-+H^++e^-$
7	$\frac{1}{2}HS^-\rightarrow\frac{1}{2}H^++\frac{1}{2}S+e^-$
8	$\frac{1}{20}C_5H_7O_2N+\frac{9}{20}H_2O\rightarrow\frac{1}{5}CO_2+\frac{1}{20}HCO_3^-+\frac{1}{20}NH_4^++H^++e^-$

Table S8. Representation of how different groups in Table S5 are combined to create different DNRA and denitrification scenarios. The index 1 shows that a group is present in a scenario and the index 0 shows that it is absent from the scenario.

DNRA					
Status	Scenario	A	B	C	D
Valid	A	1	0	0	0
Valid	AB	1	1	0	0
Valid	AC	1	0	1	0
Not valid*	AD	1	0	0	1
Valid	ABC	1	1	1	0
Not valid*	ABD	1	1	0	1
Valid	ACD	1	0	1	1
Valid	ABCD	1	1	1	1
Valid	B	0	1	0	0
Valid	BC	0	1	1	0
Not valid*	BD	0	1	0	1
Valid	BCD	0	1	1	1
Valid	C	0	0	1	0
Valid	CD	0	0	1	1
Not valid*	D	0	0	0	1
Not valid **	-	0	0	0	0

Denitrification			
Status	Scenario	E	F
Valid	E	1	0
Valid	F	0	1
Valid	EF	1	1
Not valid **	-	0	0

- *The pair of reactions that utilize sulfur as the electron donor, cannot be present without presence of the reaction pair that oxidizes sulfide to sulfur.*

*** There is no reaction to take into account nitrate reduction*

Table S9. Constraints for kinetic parameters introduced in equation 1, used in fitting batch models and the reactive transport model

parameter	min	max
μ (1/s)	10^{-5}	10^{-2}
K_a (mol/l)	10^{-5}	10^{-2}
K_s (mol/l)	10^{-5}	10^{-2}
I (mol/l)	10^{-5}	10
$C_{biomass\ initial}^{*}$ (mol/l)	10^{-4}	0.0653

References

1. Xu, X.-j.; Chen, C.; Wang, A.-j.; Guo, H.-l.; Yuan, Y.; Lee, D.-J.; Ren, N.-q., Kinetics of nitrate and sulfate removal using a mixed microbial culture with or without limited-oxygen fed. *Applied microbiology and biotechnology* **2014,** *98*, (13), 6115-6124.
2. Hubert, C.; Nemati, M.; Jenneman, G.; Voordouw, G., Containment of Biogenic Sulfide Production in Continuous Up-Flow Packed-Bed Bioreactors with Nitrate or Nitrite. *Biotechnology progress* **2003,** *19*, (2), 338-345.
